# Thermodynamic Stability, Structure, and Optical Properties of Perovskite-Related CsPb_2_Br_5_ Single Crystals under Pressure

**DOI:** 10.1021/acs.inorgchem.2c02253

**Published:** 2022-09-01

**Authors:** Viktoriia Drushliak, Marek Szafrański

**Affiliations:** Faculty of Physics, Adam Mickiewicz University, Uniwersytetu Poznańskiego 2, 61-614Poznań, Poland

## Abstract

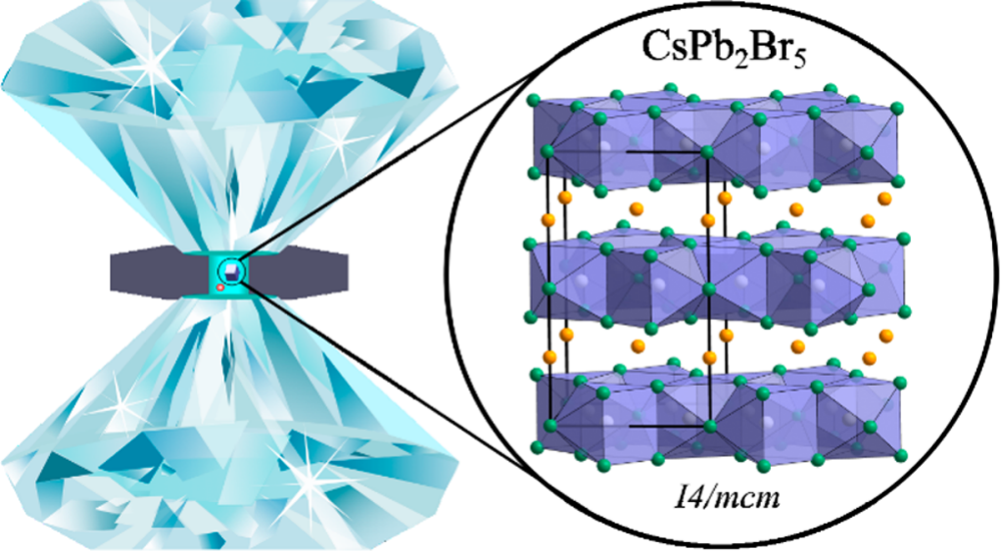

CsPb_2_Br_5_ belongs to all inorganic perovskite-related quasi-two-dimensional materials that have attracted considerable attention due to their potential for optoelectronic applications. In this study, we solve numerous controversies on the physical properties of this material. We show that optical absorption in the visible spectrum and green photoluminescence are due to microcrystallites of the three-dimensional CsPbBr_3_ perovskite settled on the CsPb_2_Br_5_ plates and that carefully cleaned crystal plates are devoid of these features. The high-pressure structural and spectroscopic experiments, performed on the single crystals free of CsPbBr_3_ impurities, evidenced that the layered tetragonal structure of CsPb_2_Br_5_ is stable at least up to 6 GPa. The absorption edge is located in the ultraviolet at around 350 nm and continuously red shifts under pressure. Moderate band gap narrowing is well correlated to the pressure-induced changes in the crystal structure. Although the compressibility of CsPb_2_Br_5_ is much higher than for CsPbBr_3_, the response in optical properties is weaker because the Pb–Br layers responsible for the optical absorption are much less affected by hydrostatic pressure than those built of Cs^+^ cations. Our study clarifies the confusing data in the literature on the optical properties and thermodynamic stability of CsPb_2_Br_5_.

## Abstract 2

The impact of genuinely hydrostatic pressure on the structure and optical properties of pure CsPb_2_Br_5_ was studied for the first time on single crystals. The results provide new information about the crystal stability, photoluminescence, and optical absorption.

## 1 Introduction

In the recent decade, organic–inorganic perovskite semiconductors have attracted unprecedented attention from the scientific community.^1^ This interest stems from their excellent photovoltaic and optoelectronic properties combined with easy and low-cost fabrication. However, the long-term stability problems in the operating environment have not yet been solved. In this area, a very promising research direction is connected with all-inorganic perovskites that exhibit better resistance to atmospheric conditions, while their optoelectronic parameters are comparable to those of their hybrid counterparts, as was demonstrated for CsPbBr_3_.^2^ Cesium lead bromide can crystallize in orange (three-dimensional, 3D) and white (one-dimensional, 1D) forms, but also with different stoichiometries,^3^ forming low-dimensional structures: Cs_4_PbBr_6_, often termed a zero-dimensional perovskite (0D), and CsPb_2_Br_5_, exhibiting a two-dimensional architecture (2D). These low-dimensional perovskite-related materials have gained remarkable interest because of strong photoluminescence (PL) in the visible spectrum,^4,5^ reported recently, which opens prospects for applications in light-emitting diodes,^6^ lasers,^7^ and photodetectors.^8,9^

**Figure 1:**
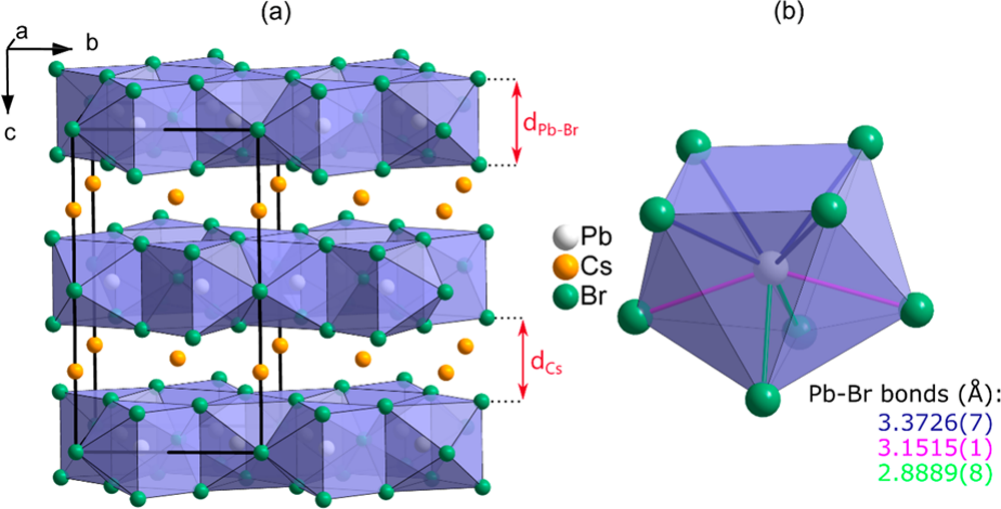
(a) Crystal structure of CsPb_2_Br_5_ with the thickness of the Pb–Br and Cs layers marked by the arrows and (b) the bicapped trigonal prism PbBr_8_ being the building unit of the Pb–Br layers. The three different Pb–Br distances and their values determined under ambient conditions are marked with different colors.

Here, we present a systematic structural and optical spectroscopic high-pressure study performed on pure single crystals of CsPb_2_Br_5_. In the tetragonal structure of CsPb_2_Br_5_, the bicapped trigonal prisms PbBr_8_ are connected by faces into layers perpendicular to the *c*-axis. These Pb–Br layers are sandwiched between layers composed of Cs^+^ cations.^10,11^ The crystal structure is shown in Figure 1. The quasi-2D character of the structure should result in a wider band gap compared to that of the 3D orange form of CsPbBr_3_ for which the energy gap *E*_g_ = 2.34 eV^12^ (λ = 530 nm). Indeed, in the original work,^3^ published by Wells in 1893, CsPb_2_Br_5_ is characterized as a white powder, indicating an absorption edge in the ultraviolet region. Therefore, the location of the absorption edge in the visible spectrum around 515–525 nm in numerous papers^5,6,8,13^ is highly questionable because in that case the crystal could not be colorless. Furthermore, theoretical calculations of the electronic structure indicate that the energy gap in CsPb_2_Br_5_ is of indirect type,^5,10,14^ and for that reason, strong PL should be excluded. In fact, detailed studies of carefully prepared material showed that pure CsPb_2_Br_5_ does not absorb light in the visible region and is PL inactive.^10,14,15^ The controversial strong green emission was discussed by several groups, which indicated possible origins of this emission, such as impurities of the orange phase of CsPbBr_3_ mixed with CsPb_2_Br_5_,^16^ CsPbBr_3_ nanocrystals embedded in CsPb_2_Br_5_ microplates,^17^ amorphous lead bromide ammonium complexes present on the surface of the nanosheets,^18^ or intrinsic crystal defects.^19^ It appears that the methodology of CsPb_2_Br_5_ preparation is crucial in this respect because even a small amount of CsPbBr_3_ admixture strongly affects the optical properties of the material obtained. This is clearly seen in previously published studies,^5,6,8,13^ where on the basis of the standard powder X-ray diffraction analysis the material is defined as “pure”, while its absorption edge and PL properties are evidently dominated by the presence of the orange form of CsPbBr_3_, and they are dramatically different from those of the truly pure substance.^10,15^ Although from the point of view of optoelectronic applications the strongly light-emitting two-phase material is very attractive, the physical properties determined for such material and their assignment to the pure form lead to misleading information and errors of interpretation of the data.

In this study, we report the response of the CsPb_2_Br_5_ structure to hydrostatic pressure and the related changes in the optical properties of the crystal. Special emphasis was placed on the synthesis and crystallization procedures. We show that our results obtained for pure single crystals are completely different from those published previously.^13^

## 2 Materials and Methods

### 2.1 Chemicals

Cesium carbonate (Aldrich, 99%), hydrobromic acid for analysis (Acros Organics, 48% solution in water), and lead(II) acetate trihydrate (Aldrich, 99.999%) were purchased and used without further purification. The water was purified by double distillation.

### 2.2 Synthesis of CsPb_2_Br_5_

Cesium bromide and lead(II) bromide were obtained by reactions of hydrobromic acid with cesium carbonate and lead(II) acetate trihydrate. The stoichiometric amounts of CsBr and PbBr_2_ were dissolved in hot water acidified with HBr. Slow cooling of the solution to room temperature resulted in the crystallization of thin CsPb_2_Br_5_ plates. The precipitated crystals were filtrated, washed with hexane, and dried. We also performed a series of crystallizations to determine the optimal conditions for growing the good-quality pure crystals. In these experiments, the amount of HBr acid in the solution and/or the CsBr:PbBr_2_ ratio were modified.

### 2.3 Calorimetric and Thermogravimetric Analysis

The thermal stability of CsPb_2_Br_5_ was studied by the thermogravimetric analysis method (TGA) using a TGA Q50 instrument (TA Instruments) and by differential scanning calorimetry (DSC) with a Q2000 calorimeter (TA Instruments). DSC experiments were performed on as-grown crystals while TGA runs were measured on powdered samples, both at a temperature change rate of 10 K min^–1^.

### 2.4 Single-Crystal High-Pressure X-ray Diffraction

A Merrill-Bassett diamond anvil cell (DAC)^20^ equipped with diamond anvils (type Ia, 800 μm culets) supported on steel discs with conical windows was used for single-crystal X-ray diffraction (SCXRD) high-pressure experiments. The single crystal of CsPb_2_Br_5_ and the ruby chip for pressure calibration were glued to the culet of one anvil and placed into the spark-eroded hole in the tungsten gasket (initial thickness 250 μm, hole diameter 350–380 μm). The chamber was filled with isopropanol as a pressure medium, ensuring hydrostatic conditions up to ∼4.2 GPa.^21^ The pressure inside the DAC was monitored by using the ruby fluorescence method,^22^ before and after each measurement. The overall pressure uncertainty was <0.03 GPa. All SCXRD measurements were performed at room temperature by using an Oxford Diffraction Gemini A Ultra diffractometer operating with graphite-monochromated Mo Kα radiation (λ = 0.71073 Å). Data were collected and processed by using CrysAlisPro software.^23^ The structures were solved and refined by using SHELX programs.^24^ Crystallographic information files (CIFs) for CsPb_2_Br_5_ structures determined under different pressures (2176587–2176600) have been deposited in the Inorganic Crystal Structure Database.

### 2.5 Optical Measurements

For high-pressure optical spectroscopic measurements, diamond anvils type IIa, supported by tungsten carbide seats with conical windows, were used. The hydrostatic liquid and other experimental details were the same as in the case of diffraction experiments. The pressure dependence of the absorption edge was measured on a 3.9 μm thick plate and on a nanosheet of the average thickness 30 nm, which was grown in situ on the diamond culet surface. The thickness of the nanosheet was determined with the atomic force microscopy (AFM) technique, whereas an interference method combined with optical microscopy was used to measure the thickness of the microplates (see Figure S1). Absorption and diffuse reflectance spectra were recorded by a Jasco MSV-5100 microscopic spectrophotometer. The absorption spectra were collected from the small area of the crystals of 30 μm in diameter by using a continuous speed of 200 nm min^–1^ and a spectral bandwidth of 5 nm.

PL spectra were measured with a homemade attachment to the Jasco MSV-5100 spectrophotometer, where a xenon lamp served as an excitation source in the spectral range 320–500 nm, and a Spectra Academy SV2100 spectrometer was applied for analysis of the spectrum emitted by the sample. All spectroscopic measurements were performed at room temperature.

## 3 Results and Discussion

### 3.1 Characterization of the Material

**Figure 2:**
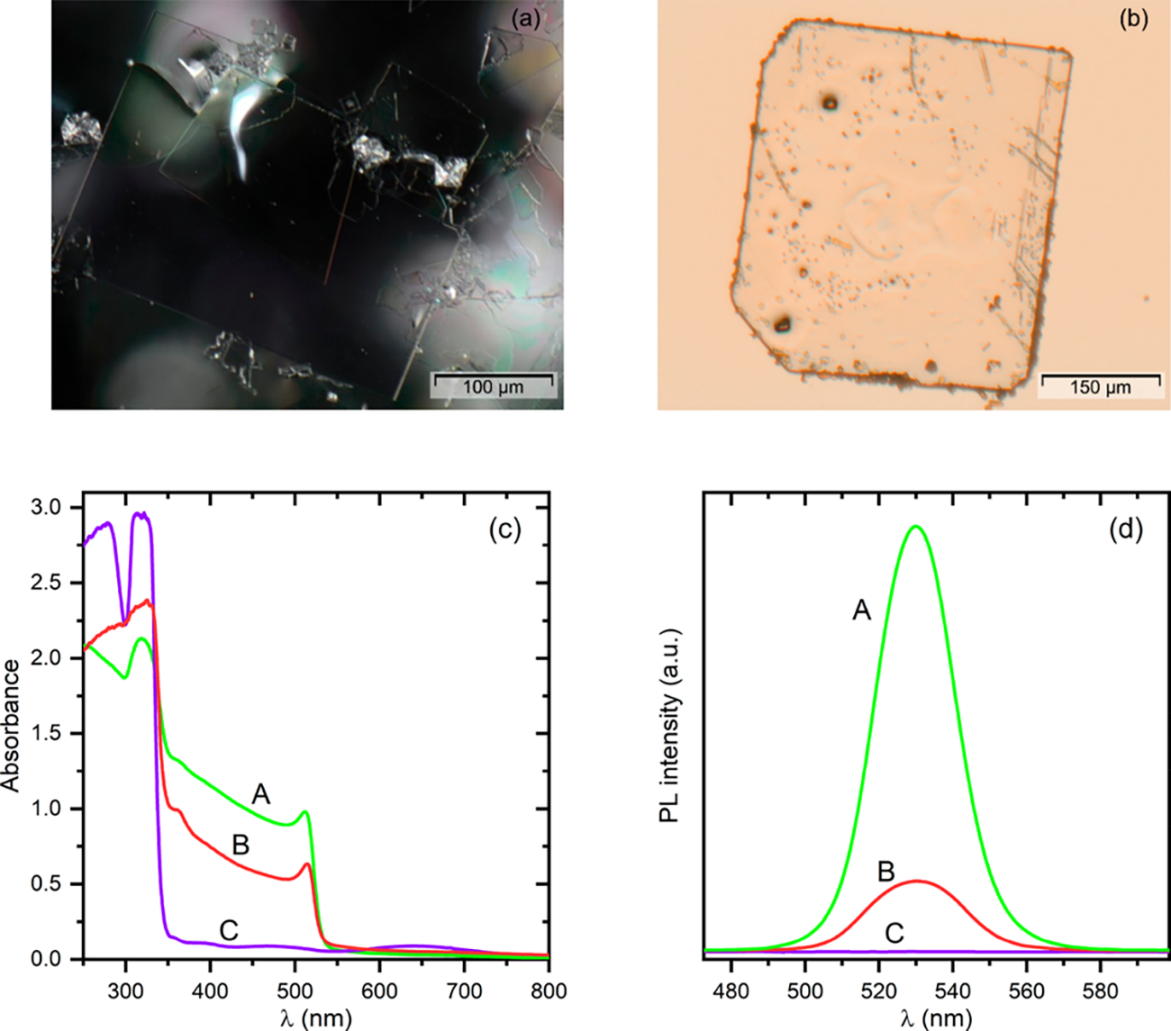
Photographs of crystals taken from the solution: (a) after washing and drying (reflected light) and (b) after drying only, with visible microcrystals of orange CsPbBr_3_ crystallized on the surface plate (transmitted light). Absorption (c) and photoluminescence (d) spectra of CsPb_2_Br_5_. The spectra recorded for two different as-grown (unwashed) crystal plates are marked by A and B, and the spectra of the cleaned plate are marked by C.

An overview of the papers published on CsPb_2_Br_5_ shows a large diversity of methods applied for the synthesis and crystallization of this material.^3,5,6,10,14,16,25−28^ Many of these studies were focused on the preparation of microcrystals or nanocrystals by triggering off a sudden crystallization through a hot injection method.^8,13,14,17,18,25^ In such syntheses, the control of a single-phase crystallization is limited, especially when the substrates can form structures of different stoichiometries. For the purposes of this work, we used the simplest method of crystallization from an aqueous solution, which was already described by Wells in 1893^3^ and has been successfully used recently.^11,15^ The obtained crystals were colorless and of good optical quality (Figure 2a). Their layered tetragonal symmetry structure was confirmed by SCXRD measurements (Figure 1; for details, see also Table S1). The space group symmetry *I*4/*mcm* and the lattice parameters *a* = *b* = 8.4931(1) Å and *c* = 15.1786(3) Å are consistent with the previously reported data.^29^ According to our DSC and TGA measurements, this structure is stable at least between 95 and 625 K (see Figures S2 and S3). In particular, our study did not confirm the thermal anomaly observed previously at 341.5 K.^30^ The only DSC anomaly was detected at 625 K, where the crystal melts.

The purity of the crystals grown, and especially the presence of CsPbBr_3_ admixture, was monitored by the absorption spectroscopy method. The orange form of CsPbBr_3_ exhibits strong absorption below ca. 530 nm, and even trace amounts of this substance in the studied material are clearly reflected in the absorption spectrum. Therefore, this method is much more sensitive than the commonly used powder X-ray diffraction. The crystallizations performed from the water solutions, acidified with different amounts of HBr, showed that all of the obtained and carefully cleaned crystals exhibit the same optical properties; that is, they are colorless and transparent, their absorption edge is located below 350 nm, and they are devoid of green PL (see plots C in Figure 2c,d). This also concerns the crystals grown from the strongly acidified solution, which crystallized together with the orange form of CsPbBr_3_. The only visible difference was in the thickness of the plates, which clearly increased with the amount of acid in the solution. This is a result of the better solubility of the substrates and their higher concentrations in the acidified solution. It is well-known that the 3D CsPbBr_3_ perovskite crystallizes in water solution only at a sufficiently high concentration of HBr; otherwise, CsPb_2_Br_5_ is formed,^3^ regardless of the 1:1 stoichiometry of CsBr:PbBr_2_ in the solution. Our experiments confirmed that also in solutions containing an excess of CsBr the crystals of CsPb_2_Br_5_ were obtained in pure form. Thus, it is evident that CsPb_2_Br_5_ crystallizes without any inclusions of CsPbBr_3_, even in solutions of disturbed stoichiometry. However, it should be noticed that the crystals harvested from the solution have to be carefully washed and dried to remove the remaining solution. Living these remains on crystal plates leads to an increase in HBr concentration in the droplets of the mother liquor due to water evaporation, and finally it results in the precipitation of CsPbBr_3_ micro- and nanocrystallites on the surface of CsPb_2_Br_5_ crystals (Figure 2b). Therefore, such “unwashed” crystal plates absorb light in the visible spectrum below 520–530 nm and exhibit strong green PL; that is, they show typical characteristics of the orange form of CsPbBr_3_. This is illustrated in Figure 2c,d where the absorption and PL spectra of two as-grown unwashed plates (A, B) and of the cleaned plate (C) are shown. The intensity of PL is correlated to the absorbance of the samples in the spectral range 350–550 nm, which clearly depends on the amount of CsPbBr_3_ on the crystal surfaces. All our further experiments were performed on pure CsPb_2_Br_5_ crystals.

**Figure 3:**
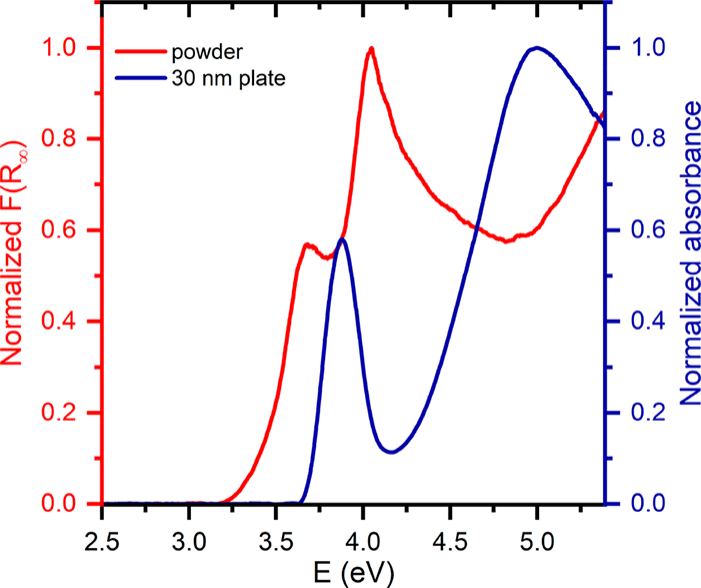
Normalized Kubelka–Munk function for powdered sample (left *Y*-axis) and normalized absorbance for 30 nm thick plate (right *Y*-axis) as functions of energy.

A review of data from the literature shows that the optical spectrum of CsPb_2_Br_5_ has been the subject of numerous studies, but only very few experiments have been performed for noncontaminated samples. In Figure 3 we compare the spectrum measured by the diffuse reflectance method with that obtained from the optical absorption measurements on the 30 nm thick sheet. The reflective spectrum is presented in the form of a normalized Kubelka–Munk function,^31^*F*(*R*_∞_) = α/*S* = (1 – *R*_∞_)^2^/2*R*_∞_, where α is the absorption coefficient, *S* is the scattering coefficient of the powdered sample, and *R*_∞_ is the diffuse reflectance of an infinitely thick layer. The two peaks visible in this spectrum at 336 nm (3.69 eV) and 307 nm (4.04 eV) are in excellent agreement with the earlier reflectance data.^11^ However, a comparison with the conventional absorption spectrum reveals essential differences, as the absorption peaks are clearly blue shifted to 320 nm (3.87 eV) and 255 nm (4.86 eV), respectively. This discrepancy can indicate a substantial difference between the bulk electronic states, responsible for the transient absorbance, and the surface electronic states that contribute mainly to the reflection. For comparison purposes, we also measured the diffuse reflectance and absorption spectra of the 3D perovskite MAPbBr_3_. These spectra, plotted in Figure S4, show the same trend as in the case of CsPb_2_Br_5_. Therefore, it is evident that the energy gaps determined from the absorption and reflectance data can differ considerably. It is worth noting that the onset of 3D lead-halide perovskites absorption is dominated by excitonic absorption,^32^ and it is also characteristic of 2D CsPb_2_Br_5_, for which the excitonic band at 320 nm determines the absorption edge of the crystal.

### 3.2 Pressure Dependence of Structural Parameters of CsPb_2_Br_5_

**Figure 4:**
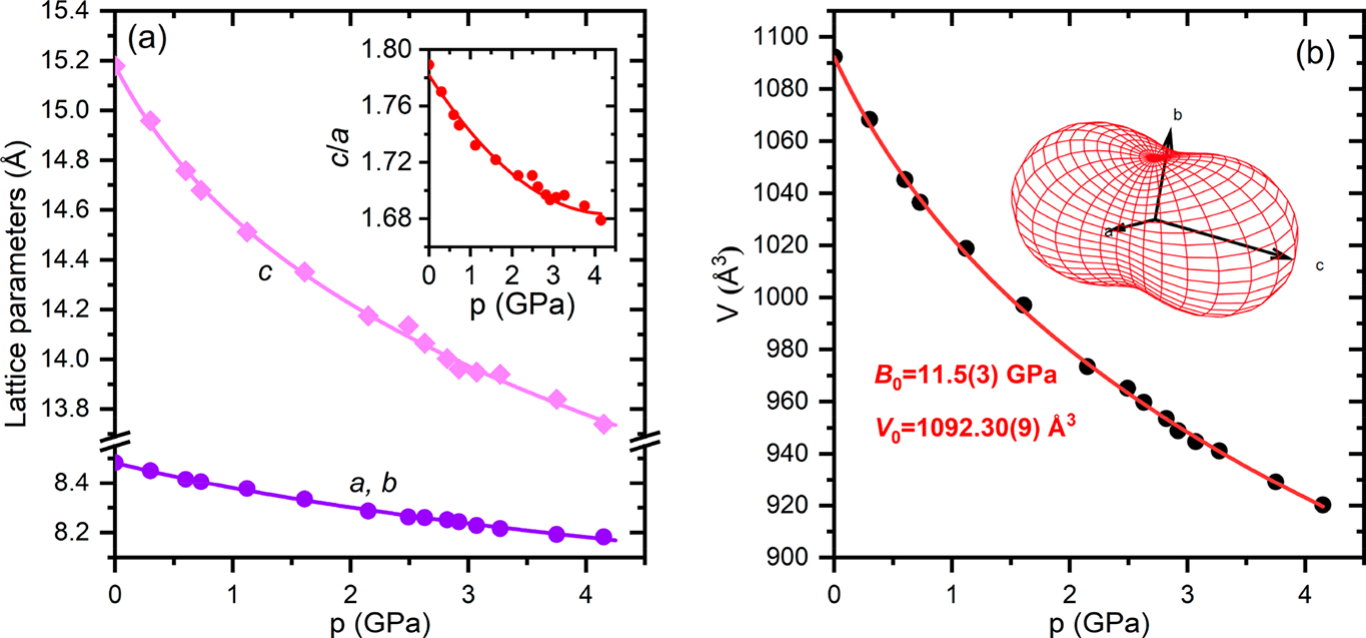
(a) Pressure dependence of the lattice parameters and (b) unit cell volume of CsPb_2_Br_5_. The lattice parameters and unit cell volume were fitted with the third-order Birch–Murnaghan EOS (solid lines). The inset in (b) shows the compressibility indicatrix calculated by using PASCAL software.^35^

The SCXRD data were collected in the pressure range to 4.2 GPa. As shown in Figure 4, the hydrostatic compression of the crystal results in a continuous shortening of the lattice parameters and a decrease in the unit cell volume. The monotonic contraction of the crystal, as well as the preservation of the space group symmetry *I*4/*mcm* in the whole studied pressure range, testifies that this layered structure is highly stable not only in a wide temperature range but also under pressure. The lack of structural transformation is surprising in contrast to the recently published high-pressure study of CsPb_2_Br_5_, where an isostructural phase transition was postulated at 1.6 GPa,^13^ associated with a stepwise change in the lattice parameters and ∼3% decrease in the unit-cell volume. However, it should be noted that in this study the measurements by powder XRD were performed by using silicon oil as a pressure medium, which is hydrostatic to ∼1 GPa only. In this case, the pressure dependence of the lattice parameters can be strongly distorted by the nonhydrostatic conditions of the experiments. For the sake of comparison, our *V*(*p*) data are plotted in Figure S5a together with those previously reported in ref (13). The most striking is a deepening discrepancy between these two data sets as the pressure increases. For example, at atmospheric pressure the unit-cell volume determined from our single-crystal data (*V* = 1092 Å^3^) is very close to that measured by the powder diffraction method in ref (13) (*V* = 1088 Å^3^), while under a pressure of 4 GPa the corresponding values strongly diverge (925 vs 965 Å^3^, respectively). Moreover, it is worth noting that the discontinuities in the lattice parameters derived from the powder diffraction data are in fact hardly correlated to the smooth changes in the presented diffraction patterns,^13^ and therefore the reported phase transition seems to be highly questionable.

The experimental *V*(*p*) data plotted in Figure 4b were fitted by the third-order Birch–Murnaghan equation of state:^33^
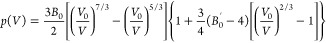
where *V*_0_ is the volume of the reference unit cell at ambient pressure, *B*_0_ is the isothermal bulk modulus at ambient pressure, and *B*_0_^′^ is the pressure derivative of the bulk modulus extrapolated to *p* = 0. The fitting procedure performed by using EosFit7-GUI program^34^ allowed us to determine the bulk modulus *B*_0_ = 11.5(3) GPa and its derivative *B*_0_^′^ = 9.3(6) of CsPb_2_Br_5_. These parameters can be compared with those of CsPbBr_3_. For this purpose, the single-crystal *V*(*p*) data published recently for the orange form of CsPbBr_3_^12^ were fitted by the third-order Birch–Murnaghan equation (see Figure S5b) with *B*_0_ = 15.0(8) GPa and *B*_0_^′^ = 6.0(18). Because *B*_0_ and *B*_0_^′^ are correlated,^36^ a comparison of these parameters for CsPb_2_Br_5_ and CsPbBr_3_ was made by plotting *B*_0_^′^ versus *B*_0_, together with their confidence ellipses, as illustrated in Figure S8. This plot shows that the confidence ellipses do not overlap even at the 99.7% confidence level, and therefore we conclude that the 3D perovskite CsPbBr_3_ is less compressible than the layered CsPb_2_Br_5_. The inset in Figure 4b shows that the compressibility of CsPb_2_Br_5_ is strongly anisotropic, much higher along the *c* direction perpendicular to the layers than in the *a–b* plane. It should be noted that this anisotropy varies with increasing pressure, as indicated by the nonlinear decrease in the *c*/*a* ratio (see the inset plot in Figure 4a). The linear compressibility coefficients calculated at zero pressure β_*a*_ = 14.3(8) TPa^–1^ and β_*c*_ = 61(3) TPa^–1^ were used to determine independently the bulk modulus *B*_0_ = 11.2(6) GPa. This value is in very good accordance with that derived from the fitting of the unit-cell volume. The low value of *B*_0_ shows that CsPb_2_Br_5_ is relatively soft as for all-inorganic material. However, this is also characteristic for 3D organic–inorganic metal halide perovskites:^37,38^ for example, for CH_3_NH_3_SnI_3_, *B*_0_ = 12.6(7) GPa; for HC(NH_2_)_2_SnI_3_, *B*_0_ = 8.0(7) GPa; and for HC(NH_2_)_2_PbI_3_, *B*_0_ = 11.0(2) GPa. The other important issue is the high value of *B*_0_^′^, which typically is close to 4 while for CsPb_2_Br_5_ it is raised to 9.3(6). This high value is justified by the layered character of the structure as similar values were reported; for example, for graphite-like BC, *B*_0_^′^ = 8.0(6),^39^ and for layered GeSe_2_, *B*_0_^′^ = 9.1(22).^40^

**Figure 5:**
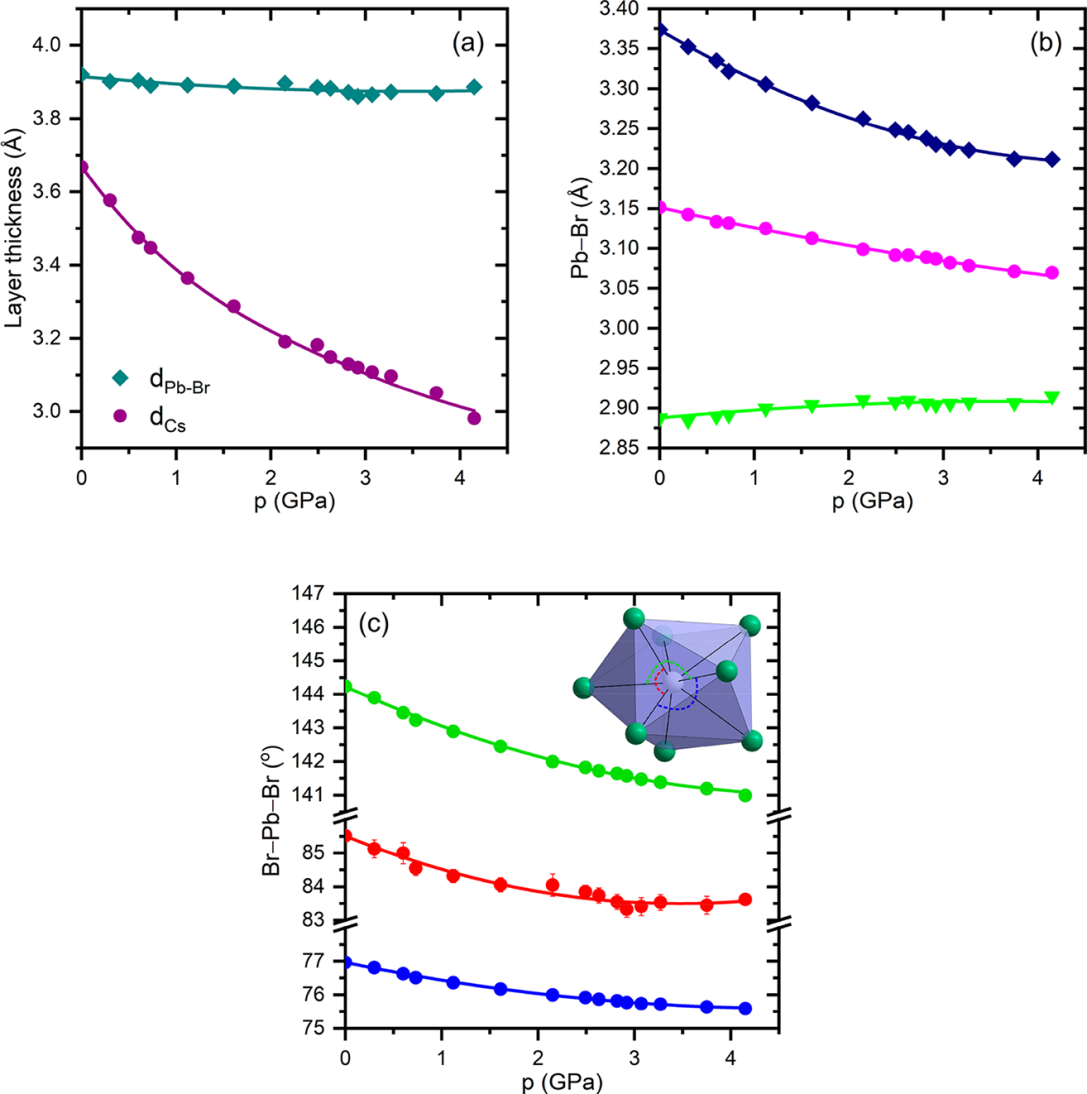
Pressure dependence of structural parameters in the compressed single crystal of CsPb_2_Br_5_: (a) the thickness of the Pb–Br and Cs layers, (b) the Pb–Br distances, and (c) the selected Br–Pb–Br angles; layers and Pb–Br distances are defined in Figure 1.

In the structure of CsPb_2_Br_5_ the layers built of the face-connected bicapped trigonal prisms PbBr_8_ are more resistant to external hydrostatic pressure than the layers composed of Cs^+^ cations. This is apparent in Figure 5a, where the changes in the thickness of both layers are plotted as a function of pressure. In the Pb–Br layers, Pb^2+^ is coordinated by eight Br^–^ that form a polyhedron with three different Pb–Br distances (see Figure 1). At atmospheric pressure, these distances are strongly differentiated taking the values 3.374 Å (4×), 3.151 Å (2×), and 2.888 Å (2×). Similarly, their pressure dependences are also diversified. As shown in Figure 5b, with increasing pressure, the six longest Pb–Br bonds contract, while the two shortest bonds slightly elongate.

### 3.3 Absorption Edge and Energy Gap of Compressed CsPb_2_Br_5_

**Figure 6:**
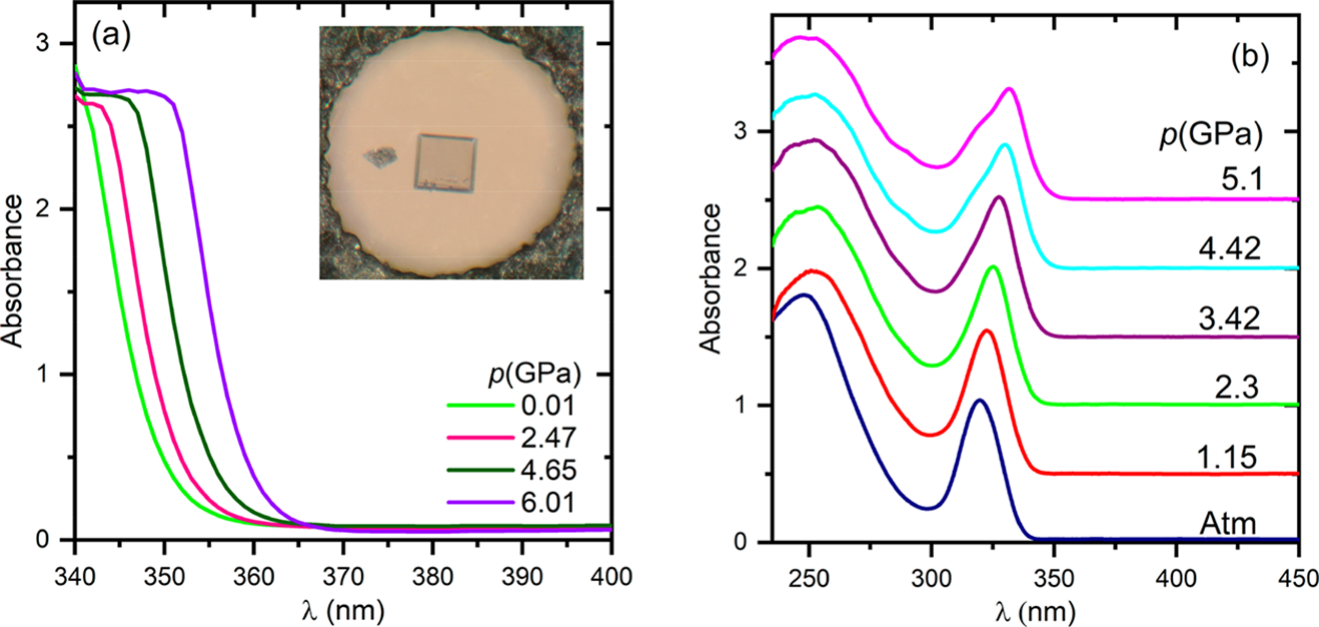
Selected absorption spectra of CsPb_2_Br_5_ recorded under different pressures: (a) for the 3.9 μm thick microplate and (b) for the 30 nm thick nanosheet. The inset shows the crystal in the DAC.

**Figure 7:**
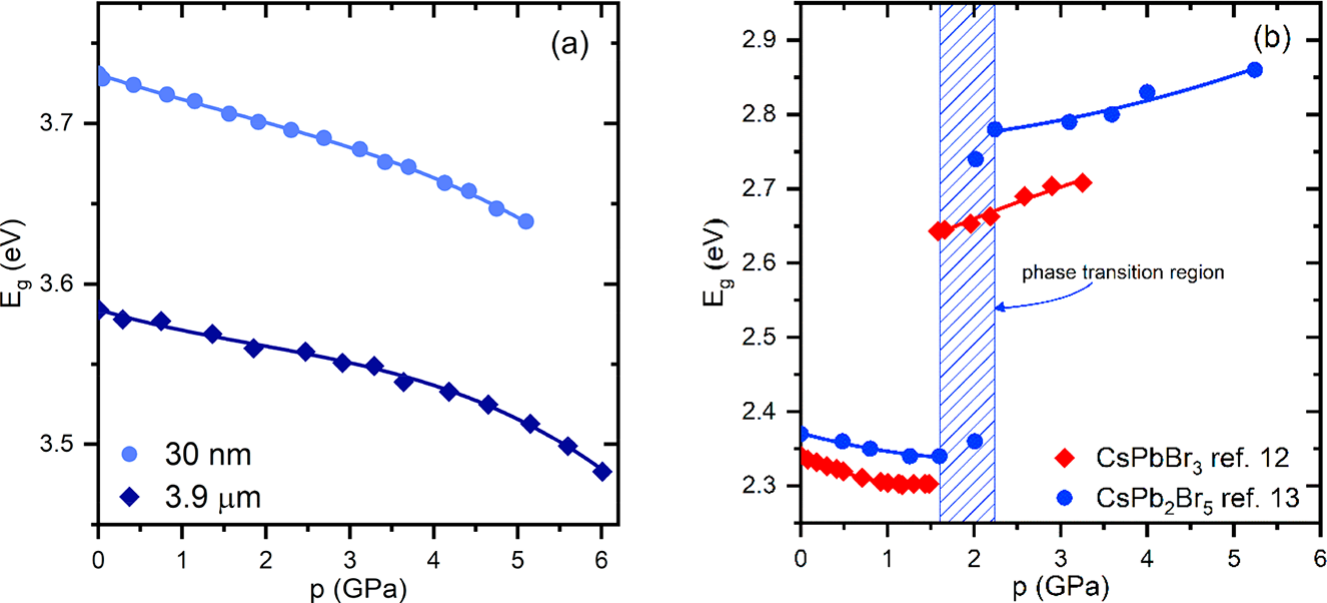
(a) Pressure-induced red shift of the band gap of CsPb_2_Br_5_ determined for two samples of different thickness in this work. (b) Comparison of the data reported in the literature for CsPb_2_Br_5_^13^ with the results published for 3D CsPbBr_3_.^12^

The absorption spectra of CsPb_2_Br_5_ were measured under pressure on a single-crystal microplate and on a nanosheet during compression cycles. The selected spectra are plotted in Figure 6. As can be seen, for the 3.9 μm thick plate only the absorption edge is accessible, while the nanosheet spectrum is structured with a well-shaped excitonic band at 320–330 nm. The absorption onset is gradually red shifted with increasing pressure. To determine the energy gap, the absorption edges were converted to Tauc plots^41^ (see Figure S6) by plotting (α*h*ν)^2^ versus photon energy *h*ν, where α is the absorption coefficient of the material. The values of *E*_g_ thus obtained are plotted as a function of pressure in Figure 7a. It is evident that the band gap of CsPb_2_Br_5_ decreases monotonically with increasing pressure, without any anomalies that could be attributed to phase transitions. The *E*_g_(*p*) dependence is linear in the pressure range to about 4 GPa, whereas under higher pressures, beyond the hydrostatic limit of isopropanol, some nonlinearity occurs. It is not clear whether this is an intrinsic feature of the crystal or the result of nonhydrostatic conditions, which can substantially affect the properties of materials.^42,43^ The character of pressure-induced changes is the same regardless of the thickness of the samples, and the only difference is that for the thicker plate the obtained value of *E*_g_ is markedly lower, but such a relation can be expected for samples of substantially different thickness, as can be deduced from the plots in Figure S7.

In the studied pressure range of 6 GPa, the overall band gap reduction is about 0.1 eV. This relatively moderate change in *E*_g_ well correlates with the subtle changes in the thickness of the compressed Pb–Br layers (Figure 5a). The calculations of the electronic band structure indicate that the band gap in CsPb_2_Br_5_ has an indirect character, but the direct gap is only slightly larger.^7^ The top valence bands are composed mainly of the Br–4p, Pb–6p and Pb–6s orbitals, while the Pb–6p and Br–4p orbitals dominantly contribute to the lowest conduction bands. The orbitals of Cs^+^ cations do not contribute to the electronic states responsible for the absorption edge, and therefore the largest pressure effect, visible in Figure 5a as a remarkable narrowing of the Cs^+^ layers, does not have a direct translation into the optical properties of the crystal. Thus, the band gap of CsPb_2_Br_5_ is mainly determined by the structure of Pb–Br layers and its modification under pressure. As shown in Figure 5b (see also Figure 1b), crystal compression results in contraction of the six longest Pb–Br distances, while the two shortest bonds slightly elongate. The shortening of Pb–Br bonds enhances the overlap of the atomic orbitals, leading to an upward shift of the maximum valence band and, as a consequence, to a red shift of the band gap. The opposite and therefore competitive effect comes from the Br–Pb–Br angles bending.^44^ The relatively small changes observed in the angles (Figure 5c) indicate that the contraction of the Pb–Br bonds has a dominant influence on the modification of the electronic states responsible for the absorption edge.

In discussing the pressure dependence of *E*_g_, we have to refer to the data reported by Ma et al.,^13^ which are plotted for comparison in Figure 7b. It is obvious that our results are in stark contrast to those obtained by Ma et al. This applies to the pressure-induced changes in the band gap as well as to its value, which is even more important. To explain these discrepancies, we plotted in Figure 7b our recently published *E*_g_(*p*) results measured for 3D CsPbBr_3_.^12^ The correlation between these data and the results presented by Ma et al. is evident. Therefore, we conclude that they studied, in fact, the pressure dependence of the CsPbBr_3_ band gap instead of CsPb_2_Br_5_. Most probably, their samples were strongly contaminated with CsPbBr_3_, and their interpretation of the results seems to be even more puzzling when considering that the same group of authors had previously published two articles on the optical properties of CsPbBr_3_ under pressure.^45,46^

## 4 Conclusions

This study confirms that green photoluminescence and absorption in the visible spectrum are not intrinsic features of CsPb_2_Br_5_ but originate from CsPbBr_3_ impurities. The microcrystallites of the orange CsPbBr_3_ do not form inclusions embedded in the crystal plates of CsPb_2_Br_5_ but precipitate on their surfaces. Therefore, we suppose that the majority of the literature data were in fact collected for the CsPb_2_Br_5_:CsPbBr_3_ composites instead of CsPb_2_Br_5_. We show also that the commonly used diffuse reflectance method for the absorption edge and band gap determination gives optical parameters that are remarkably different from those obtained from the optical absorption measurements. This discrepancy indicates that the energies of the surface and bulk electronic states of CsPb_2_Br_5_ differ significantly, which may also be characteristic for other similar materials.

Pressure was applied as a clean and effective tool to modify the distances between the atoms and bond angles and thus the related electronic structure of CsPb_2_Br_5_. Although the compressibility of this 2D perovskite-like material is larger than that of 3D CsPbBr_3_, the pressure-induced changes in the optical parameters are smaller. The layered architecture of CsPb_2_Br_5_ is reflected in a strongly anisotropic response to hydrostatic compression. The crystal shrinks much weaker in the *a–b* plane, i.e., parallel to the layers, than in perpendicular *c* direction. Furthermore, external mechanical stress affects the layers built of Cs^+^ cations much more than the Pb–Br frameworks. The moderate changes within the Pb–Br layers translate into the moderate monotonic narrowing of the band gap with increasing pressure. Moreover, our study performed on the genuinely pure crystals has shown that the tetragonal structure of CsPb_2_Br_5_ is stable in a wide temperature range, at least between 90 and 625 K, and the crystal does not undergo any structural transformation in the pressure range up to 6 GPa. These results shed new light on the physical properties of CsPb_2_Br_5_ and show the interpretative pitfalls associated with the experiments performed carelessly.

## Supporting Information Available

Selected crystallographic and refinement data, DSC and TGA results, figures illustrating experimental details, and plots illustrating a comparison to the literature data (PDF)
