# Supplementary material for: Thermodynamic Stability, Structure, and Optical Properties of Perovskite-Related CsPb2Br5 Single Crystals under Pressure
Source: Inorg Chem. 2022 Sep 1;61(36):14389–96. doi: 10.1021/acs.inorgchem.2c02253 (PMC9477227; doi:10.1021/acs.inorgchem.2c02253)
Supplement: Supplementary file 1 — ic2c02253_si_001.pdf [file ic2c02253_si_001.pdf]

Supporting Information

Thermodynamic Stability, Structure and Optical Properties of Perovskite-Related $CsPb_2Br_5$ Single Crystals Under Pressure

Viktoriia Drushliak and Marek Szafrański*

Faculty of Physics, Adam Mickiewicz University, Uniwersytetu Poznańskiego 2, 61-614 Poznań, Poland

Corresponding author e-mail: masza@amu.edu.pl

Table S1. Selected crystallographic and refinement data for the structures of $CsPb_2Br_5$.

Crystal formula	$CsPb_2Br_5$					
Crystal system<br>Space group<br>Z	Tetragonal<br>*I*4/*mcm*<br>4					
Pressure (GPa)	0.0001	0.73	1.12	2.15	3.07	3.75
Crystal size (mm)	0.16 × 0.12 × 0.09	0.21 × 0.21 × 0.03	0.23 × 0.15 × 0.02	0.21 × 0.21 × 0.03	0.23 × 0.15 × 0.02	0.23 × 0.15 × 0.02
a,b (Å)<br>*c* (Å)	8.4831 (1)<br>15.1786 (3)	8.4043(7)<br>14. 675(2)	8.3789 (4)<br>14.512 (12)	8.2869 (3)<br>14.175 (14)	8.2293 (6)<br>13.949 (13)	8.1935 (9)<br>13.84 (2)
Volume (Å^3)	1092.30 (3)	1036.5(2)	1018.9 (8)	973.4 (10)	944.7 (9)	929.1 (15)
ρ (g cm^{-3})	5.758	6.068	6.173	6.461	6.658	6.769
μ (mm^{-1})	52.25	55.10	56.06	58.67	60.46	61.47
No. of measured, independent and observed [$I > 2\sigma(I)$] reflections	6625, 451, 433	2200, 207, 165	2178, 134, 133	2146, 117, 108	2282, 136, 128	2233, 137, 130
R_{int}	0.055	0.066	0.078	0.103	0.094	0.091
R_1 [$I > 2\sigma(I)$], $R_1(all)$	0.020, 0.021	0.034, 0.048	0.024, 0.024	0.022, 0.028	0.032, 0.035	0.029, 0.030
wR_2 [$I > 2\sigma(I)$], wR_2 (all)	0.086, 0.088	0.081, 0.084	0.060, 0.060	0.057, 0.058	0.082, 0.084	0.071, 0.072
S	0.756	1.151	1.273	1.354	1.250	1.207

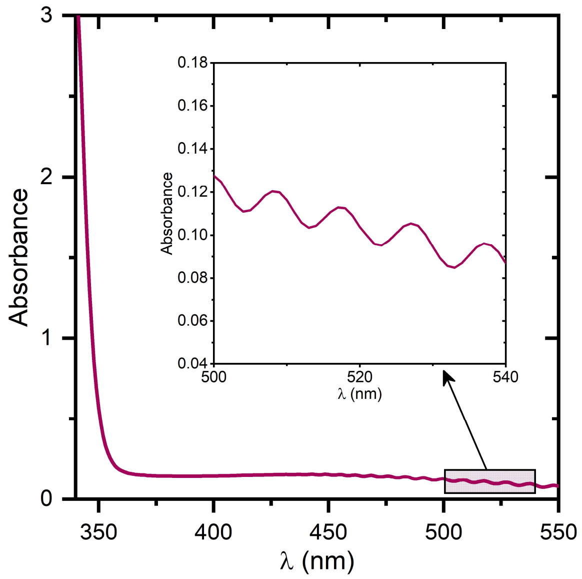

Figure S1. Interference fringes observed outside the absorption region of the $CsPb_2Br_5$ crystal plate. The positions of the interference maxima/minima were used to determine the thickness of the crystal.

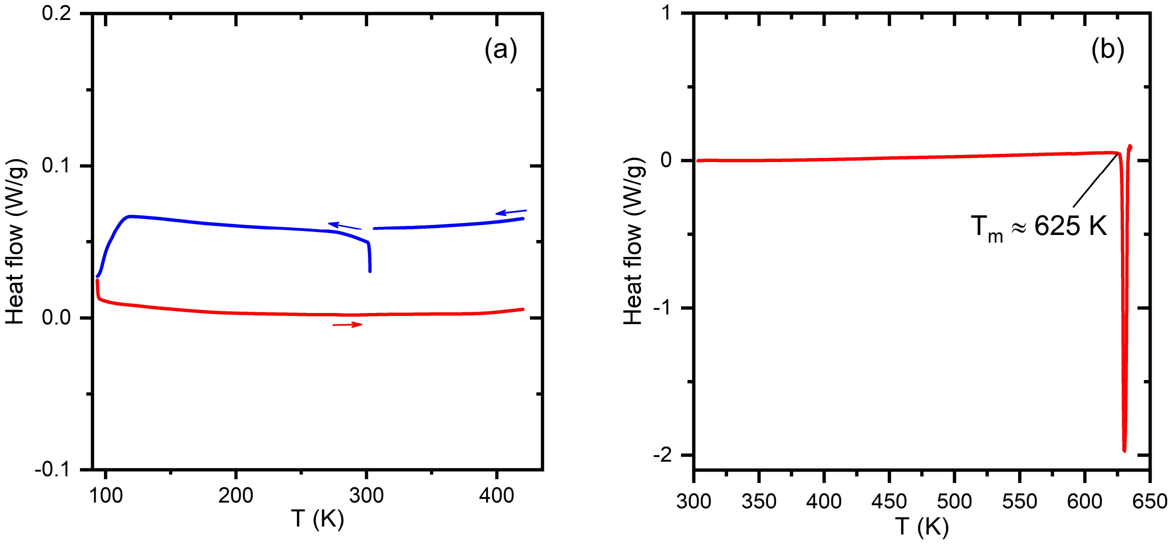

Figure S2. DSC runs recorded for $CsPb_2Br_5$ in the temperature range 95–420 K (a) and in the high-temperature range close to the melting point (b). The temperature was varied at a rate of 10 $Kmin^{-1}$.

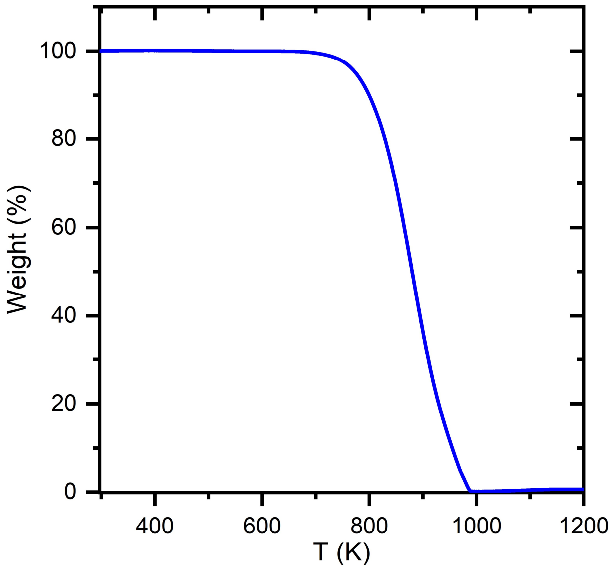

Figure S3. TGA curve for powdered $CsPb_2Br_5$ measured at a rate of 10 $Kmin^{-1}$.

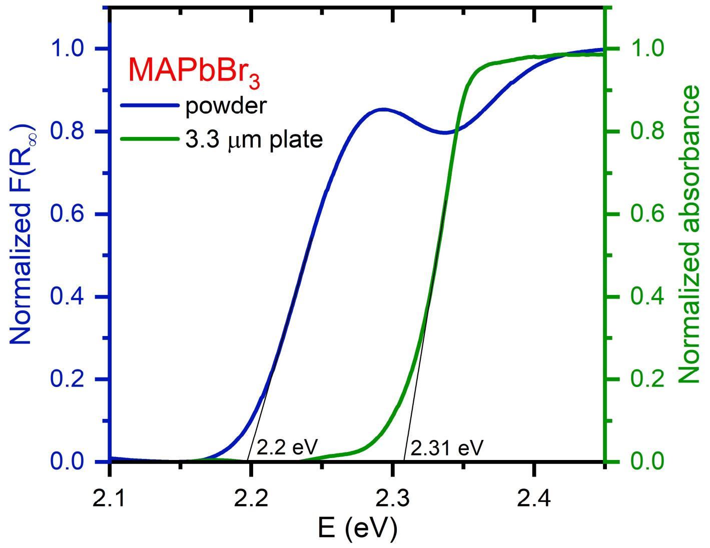

Figure S4. Comparison of the absorption and diffuse reflectance spectra for the methylammonium lead tribromide, $MAPbBr_3$.

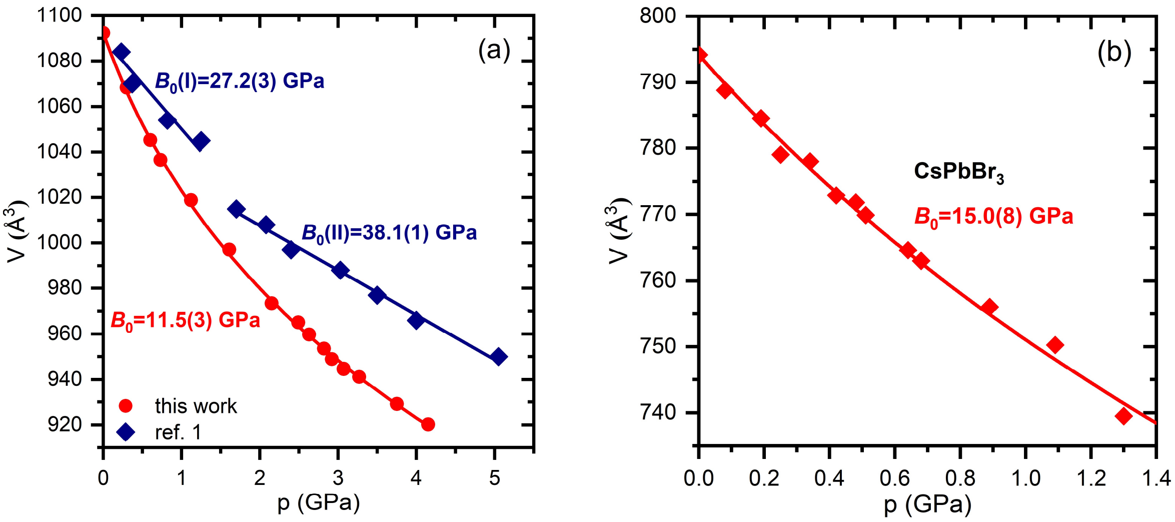

Figure S5. (a) Pressure dependence of the unit cell volume of $CsPb_2Br_5$ determined in this work from the single-crystal X-ray diffraction and compared to the literature data measured by powder diffraction.[1] The solid lines correspond to the fitted equation of states. (b) The literature *V*(*p*) data[2] fitted with the second-order Birch-Murnaghan equation of state.

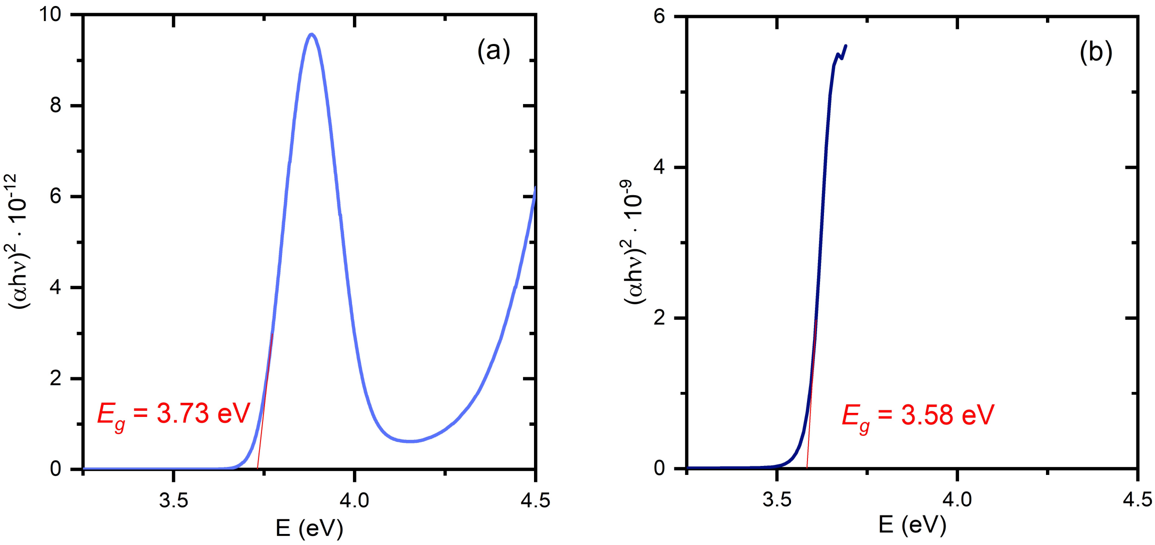

Figure S6. Tauc plots[3] and energy gaps determined from the absorption spectra measured for the 30 nm (a) and 3.9 μm (b) thick $CsPb_2Br_5$ plates.

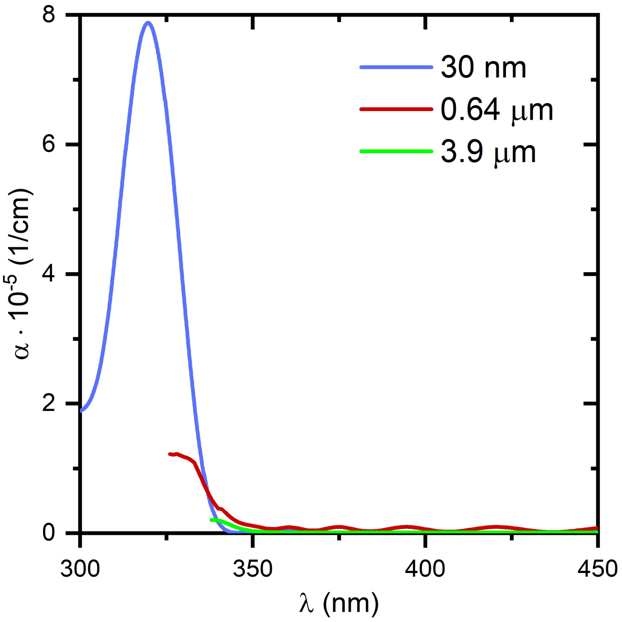

Figure S7. Absorption spectra of $CsPb_2Br_5$ measured for the plates of different thickness.

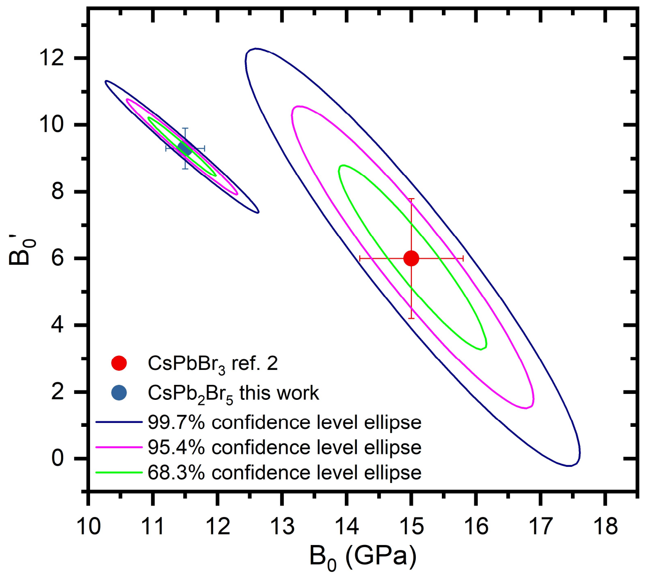

Figure S8. Values B_0' plotted versus B_0 for $CsPb_2Br_5$ and $CsPbBr_3$. The different confidence level ellipses are shown.

REFERENCES

(1) Ma, Z.; Li, F.; Qi, G.; Wang, L.; Liu, C.; Wang, K.; Xiao, G.; Zou, B. Structural Stability and Optical Properties of Two-Dimensional Perovskite-like $CsPb_2Br_5$ Microplates in Response to Pressure. *Nanoscale* **2019**, *11*, 820–825.

(2) Szafrański, M.; Katrusiak, A.; Ståhl, K. Time-Dependent Transformation Routes of Perovskites $CsPbBr_3$ and $CsPbCl_3$ under High Pressure. *J. Mater. Chem. A* **2021**, *9*, 10769–10779.

(3) Tauc, J.; Grigorovici, R.; Vancu, A. Optical Properties and Electronic Structure of Amorphous Germanium. *Phys. Status Solidi B* **1966**, *15*, 627–637.
